# Simultaneous enhancement of biogas production and antibiotic resistance gene removal by sawdust biochar in anaerobic digestion of chicken manure

**DOI:** 10.3389/fbioe.2026.1897972

**Published:** 2026-10-21

**Authors:** Xiaomei Jiang, Ting Shen, Yunhui Lei, Xiaobo Guo

**Affiliations:** 1 Biogas Institute of Ministry of Agriculture and Rural Affairs, Chengdu, China; 2 Key Laboratory of Development and Application of Rural Renewable Energy, Ministry of Agriculture and Rural Affairs, Chengdu, China; 3 Research Center for Rural Energy and Ecology, Chinese Academy of Agricultural Sciences, Chengdu, China

**Keywords:** anaerobic digestion, antibiotic resistance genes, biochar, high-ammonia chicken manure, microbial communities

## Abstract

This study evaluated the impacts of two types of biochar, namely, corn-stover and sawdust, on biogas yield and the removal of antibiotic resistance genes (ARGs) during the anaerobic digestion (AD) of chicken manure. Corn-stover biochar and sawdust biochar maintained the pH under neutral conditions, and simultaneously, effective hydrolysis, acidification, acetogenesis, and methanogenesis avoided excessive volatile fatty acids (VFAs) and free ammonia (FAN) inhibition, resulting in a 27.69% (corn-stover biochar) and 19.65% (sawdust biochar) increase in cumulative gas production. However, the low specific surface area of sawdust biochar was not conducive to the growth and reproduction of microorganisms, reducing the total number of microorganisms and thus enhancing the removal of macrolide and sulfonamide ARGs’ absolute abundance compared with corn-stover biochar. Microbial community analysis revealed that biochar addition enriched *Methanosarcina* (up to >96% relative abundance) and syntrophic bacteria such as *Sphaerochaeta* and *Fastidiosipila*, which facilitated methanogenesis and substrate degradation. Furthermore, structural equation models (SEMs) revealed that total ammonia nitrogen (TAN), pH, and FAN indirectly determine ARG abundance by influencing potential hosts and *intI1*. This study can provide a theoretical basis for the application of these common biochars in enhancing the efficiency of chicken manure AD and reducing environmental ARGs.

## 1 Introduction

Annually, approximately 155 million tons of chicken manure are generated in China (NDRC, 2017), necessitating effective disposal methods. Heavy metals, antibiotics, and antibiotic resistance genes (ARGs) are the main harmful substances in chicken manure; especially, the spread of ARGs has been receiving more and more attention (Congilosi and Aga, 2021; Gržinić et al., 2023). ARGs can be disseminated via the horizontal gene transfer pathway of mobile genetic elements (MGEs); the field application of chicken manure as organic fertilizer can release a substantial amount of ARGs into soil ecosystems (Gržinić et al., 2023), which poses a risk to human health. Previous studies have found that there were at least 50 subtypes with high abundance in chicken manure (Cui et al., 2016; Zhao et al., 2021). Therefore, an urgent environmental problem to be solved in the utilization of chicken manure is the removal of ARGs (Congilosi and Aga, 2021; Wei et al., 2026).

Anaerobic digestion (AD) is a widely recognized and environmentally sustainable method to produce organic fertilizer and methane (CH_4_) that can be used as renewable energy (Uddin and Wright, 2023; Sun et al., 2018; Zhang et al., 2019), and it is encouraging that anaerobic digestion could also contribute to the removal of ARGs (Jang et al., 2018; Lee et al., 2020; Yun et al., 2021). However, it is important to note that the removal degree of ARGs during AD is very limited (Pang et al., 2022), which may enhance the environmental pollution risk of subsequent utilization of by-products. Therefore, improving the performance of AD systems to increase the removal efficiency of ARGs is crucial. The addition of carbon-based conductive materials into AD systems can have simultaneously positive effects on AD performance and the fate of ARGs, which have a double benefit (Cai et al., 2022; Wei et al., 2020). There is growing interest in the use of biochar in AD due to its current role in the circular economy (Li et al., 2026; Wang et al., 2025). Biochar is a low-cost additive with many beneficial properties, such as a highly developed porous structure, oxygen-containing functional groups, and extensive specific surface area (SSA) (Fang et al., 2022; Li et al., 2020; Qian et al., 2026). The SSA not only provides space for microbial growth but also accelerates direct electron transfer (Li et al., 2022; Wang et al., 2021; Zhao K et al., 2024). According to Li et al. (2022), biochar significantly reduced the abundance of aadA, ant (2″)–I, qacEdelta1 and *sul1*. Yang et al. (2022a) showed that biochar effectively inhibited the horizontal transfer of ARGs. According to Yang et al. (2021), rice husk biochar indirectly reduced the ARG abundance by influencing the class 1 integron integrase gene (*intI1*).

Chicken manure is a typical high-nitrogen livestock waste, and severe ammonia accumulation readily occurs in anaerobic digestion (AD) systems (Ngo et al., 2022). Reports indicate that ammonia inhibition can result in biogas yield losses of up to 30%–60% in full-scale AD facilities (Capson-Tojo et al., 2020; Di et al., 2022). Accordingly, ammonia inhibition represents the most critical bottleneck restricting the large-scale application of AD for chicken manure valorization, which urgently needs to be addressed to facilitate efficient bioenergy recovery. Organic nitrogen contained in feedstock is mineralized to inorganic nitrogen during the hydrolytic phase of AD, which predominantly exists in digested slurry in two forms: ammonium ions and free ammonia (FAN). Owing to its lipophilic property, FAN readily penetrates microbial cell membranes. For this reason, FAN has been universally acknowledged as the dominant toxic species triggering ammonia inhibition (Han et al., 2026). Recently, some studies have explored the efficacy of biochar application in mitigating ammonia nitrogen accumulation during chicken manure anaerobic digestion, verifying a significant positive correlation between biochar supplementation and TAN reduction (Xiao et al., 2025; Ma et al., 2021; Pan et al., 2019).

Although numerous studies have verified that biochar can effectively enhance anaerobic digestion performance, most previous investigations primarily focused on biogas production improvement while neglecting the coupled variation of antibiotic resistance genes (Zhao et al., 2021). Interestingly, certain ARG-hosting microorganisms are functionally active in organic matter degradation and acid fermentation; accordingly, the survival and enrichment of specific ARG-carrying microbes can partially facilitate substrate hydrolysis and volatile fatty acid (VFA) metabolism, thereby contributing to improved biogas productivity (Shan et al., 2026). Furthermore, how different biochar properties mediate microbial community succession and further regulate ARG removal remains poorly understood.

Corn-stover biochar and sawdust biochar were selected as representative agricultural waste-derived biochars with distinctly different physicochemical properties. As shown in Table 1, their BET specific surface areas differ by approximately 18-fold (44.85 vs. 2.46 m^2^/g), whereas their pore volumes and pore sizes also exhibit marked contrasts. This substantial divergence in SSA, although their feedstock origins (both being lignocellulosic biomass) remain comparable, provides an ideal model system to isolate and investigate the specific role of biochar surface structural properties in modulating microbial colonization, DIET efficiency, and consequently, ARG fate during AD. Moreover, both biochars are low-cost and abundantly available in China, enhancing the practical relevance of this study.

**TABLE 1:** Properties of chicken manure, wheat straw and biochar.

Content	Chicken manure	Inoculum	Wheat straw	Sawdust biochar	Corn-stover biochar
pH	6.23 ± 0.21	7.32 ± 0.13	7.44 ± 0.42	7.80 ± 0.60	6.79 ± 0.15
TS (%*ww*)	22.10 ± 0.50	8.01 ± 0.91	93.83 ± 9.45	96.50 ± 6.21	94.10 ± 10.31
VS (%*ww*)	19.11 ± 0.02	2.08 ± 0.05	–	–	–
Moisture (%)	77.90 ± 3.10	91.99 ± 1.31	6.17 ± 0.45	3.50 ± 0.21	5.90 ± 0.31
TC (g/kg)	375.10 ± 8.08	–	560.45 ± 10.11	95.56 ± 7.21	93.21 ± 1.56
TN (g/kg)	39.80 ± 2.90	–	7.33 ± 0.29	–	–
BET surface area (m^2^/g)	–	–	–	2.46 ± 0.27	44.85 ± 0.34
Micropore volume (cm^3^/g)	–	–	–	0.0020 ± 0.0008	0.015 ± 0.005
Pore size (nm)	–	–	–	2.95 ± 0.16	1.85 ± 0.13

In this study, two widely available agricultural biochars (corn-stover biochar and sawdust biochar) with contrasting SSA values were selected to elucidate their divergent effects on biogas production and ARG attenuation under high-ammonia conditions. The specific objectives were (1) to evaluate the effects of biochar type on cumulative biogas yield and AD stability parameters (pH, VFAs, TAN, and FAN); (2) to quantify the absolute abundance variations of 12 representative ARGs (macrolide, sulfonamide, and tetracycline) and the mobile genetic element intI1; (3) to unravel the microbial community succession patterns and identify potential ARGs hosts; and (4) to establish a structural equation model (SEM) revealing the direct and indirect pathways through which environmental factors (TAN, pH, and FAN) regulate ARG fate. Importantly, this study systematically reveals the intrinsic correlation between biochar specific surface area, microbial population variation, and subtype-dependent ARG attenuation, providing novel guidance for the targeted selection of biochar materials to balance energy recovery and environmental risk control in manure anaerobic digestion.

## 2 Materials and methods

### 2.1 Materials

Chicken manure was sourced from a poultry farm located in the Chengdu District of Sichuan, China. The inoculum (i.e., anaerobically digested sludge) was collected from a biogas plant with chicken manure as feedstock on the same farm. Sawdust and corn-stover biochar were purchased from a biochar company in Hangzhou (Zhejiang, China), which employs pyrolysis at 550 °C to produce biochar. In addition, wheat straw was cut and crushed to 0.10–0.50 mm to achieve a C/N weight ratio of approximately 25, thereby facilitating a rapid initiation of AD. The properties of chicken manure, wheat straw, and biochar are detailed in Table 1.

### 2.2 Experimental design

Nine glass bottles, each with a total volume of 1,000 mL, were utilized as AD reactors and were categorized into three groups, each consisting of three replicates: C (no biochar added), CB (corn-stover biochar added), and SB (sawdust biochar added). First, 130 g of chicken manure and 20 g of wheat straw were introduced into each glass bottle to achieve a C/N ratio of approximately 25 for the substrate (Wang et al., 2014). Subsequently, the inoculum and distilled water were incorporated to adjust the total solids content in the AD reactors to 6% (w/v). A series of preliminary trials with biochar dosages of 1%, 5%, and 10% were conducted in advance. The experimental results demonstrated that the reactor amended with 5% biochar exhibited the optimal overall anaerobic digestion performance in terms of methane yield, pH stability and VFA degradation efficiency. Accordingly, a biochar dosage of 5% was selected for the formal test in this study. A unified biochar dosage of 5% was applied to both CB and SB treatments to ensure a fair comparison of their intrinsic material effects by eliminating dosage as a confounding variable. Moreover, the 5% dosage was supported by preliminary trials and falls within the optimal range (1%–10% w/v) commonly reported for manure-based AD systems (Cai et al., 2022; Ngo et al., 2024; Pan et al., 2019). Finally, 5% (w/v) corn-stover biochar and sawdust biochar were added to the CB reactor and SB reactor, respectively.

The headspace air in the nine AD reactors was replaced with nitrogen and maintained in a water bath at a constant temperature of 35 °C ± 0.1 °C. Each glass bottle was connected to a water-filled collection apparatus via a latex tube, and the volume of water displaced from the collection cylinder was measured to determine the volume of biogas produced. The airtightness of these devices was carefully checked. The AD experiment was conducted for 45 days until no biogas was produced.

### 2.3 Analytical methods

Fourier-transform infrared spectroscopy (FT-IR) analysis was conducted using a Thermo Fisher Nicolet 1s10 to identify the functional groups of biochar. The nitrogen adsorption–desorption isotherm at 77 K was measured by a Micromeritics instrument (Micromeritics, United States), on which Brunauer–Emmett–Teller (BET) surface areas of the biochar were calculated. Additionally, the elemental composition of the biochar was analyzed with an elemental analyzer (Elementar, Germany) to evaluate the physical and chemical properties of the two biochars. Moreover, the concentrations of ammonia nitrogen and volatile fatty acids (VFAs) were assessed to evaluate the stability of AD. The concentrations of TAN, VFAs, and free ammonia (FAN) were measured according to Xiao et al. (2019). Notably, TAN was defined as the sum of ammonium ions and free ammonia (FAN). The soluble chemical oxygen demand (SCOD) was determined to evaluate the degradation capacity of organic matter, using an LH–3C COD analyzer (Lianhua Tech. Co., Ltd., China). A pH meter (Leici, China) was used to determine the pH value.

### 2.4 Microbial community and gene quantitative analysis

Samples of the various reactors were collected in triplicate from each group on days 0, 8, 17, 33, and 45. First, total genomic DNA was extracted, and the quality and concentration of the DNA were determined according to Zhang et al. (2019). All extracted DNA was immediately stored in a −80 °C freezer for subsequent analyses of microbial community and gene quantification. The extracted DNA was analyzed using high-throughput sequencing on an Illumina platform (Illumina MiSeq PE250) to evaluate the archaeal and bacterial microbial communities. The V3-4 region of the bacterial 16S rRNA gene was amplified by PCR using the primers 338F (5′-ACT​CCT​ACG​GGA​GGC​AGC​A-3′) and 806R (5′-GGACTACHVGGGTWTCTAAT-3′). The V4-5 region of the archaeal 16S rRNA gene was amplified by PCR using the primers 524F (5′-TGYCAGCCGCCGCGGTAA-3′) and 958R (5′-YCCGGCGTTGAVTCC-AATT-3′). The PCR products were then sent to Personalbio Technology Co. Ltd., (Shanghai, China), for sequencing on the Illumina MiSeq PE250 pyrosequencing platform. The retrieved sequences were quality-filtered, merged, operational taxonomic units extracted, and clustered using QIIME2 (Xiao et al., 2019).

Since several common antibiotics, such as tetracycline, sulfonamide, and macrolide, were frequently used on farms to prevent infections, three types of ARGs targeting these antibiotics were selected and analyzed (Cui et al., 2016). The ubiquity of *intI1* in mobile genetic elements facilitates the horizontal transfer of multiple ARGs in the environment (Jang and Kan, 2022; Shao et al., 2022). Consequently, 12 ARGs (*tetG*, *tetM*, *tetQ*, *tetT*, *tetX*, *ermA*, *ermB*, *ermF*, *ermT*, *sul1*, *sul2*, and *dfrA1*), a mobile gene element *intI1*, and 16S rRNA genes were analyzed in this study. The primer set sequences and classifications of genes are given in Supplementary Table S1. Quantitative gene analysis was performed by qPCR (Bio-Rad, United States), and the specific procedures were conducted according to Zou et al. (2020).

### 2.5 Statistical analysis

Pearson correlation tests were performed using R software with the optparse and gclus libraries. The Pearson correlation coefficients were analyzed to examine the relationships among microorganism genera, ARGs, *intI1*, and various environmental factors, thereby elucidating the degree of association among these variables. Structural equation modeling (SEM) based on the correlation matrix was conducted through AMOS (SPSS Inc., Chicago, IL, United States). A structural equation model (SEM) was employed to explore the direct and indirect effects of environmental factors, microorganism genera, ARGs, and *intI1*. The mathematical and statistical calculations proposed in this paper use SPSS software (IBM SPSS Statistics 26) for data analysis.

## 3 Results and discussion

### 3.1 Performance of AD system

#### 3.1.1 Influence of biochar addition on biogas production

Figure 1a illustrates the daily biogas production profiles across the three anaerobic digestion (AD) groups. Throughout the whole digestion period, the two biochar-amended treatments (CB and SB) consistently achieved higher daily biogas yields relative to the control group (C). This stimulatory effect of biochar supplementation aligns with previous findings reporting improved methane/biogas output in chicken manure AD systems amended with wheat straw biochar, fruit wood biochar, and air-dried chicken manure-derived biochar (Ngo et al., 2024; Pan et al., 2019). Furthermore, the cumulative biogas yields reached 477.16 mL g^−1^ VS in the CB and 447.11 mL g^−1^ VS in the SB, corresponding to respective increments of 27.69% and 19.65% relative to the control C group (Figure 1b). These outcomes demonstrate that corn-stover biochar exerts a significantly stronger promotional effect on total biogas output than sawdust biochar. As listed in Table 1, the SSA of corn-stover biochar is 18-fold higher than that of sawdust biochar. Biochar with a larger SSA provides abundant attachment sites for microbial colonization and facilitates the adsorption of organic substrates, shortening mass transfer distances and accelerating biochemical metabolic reactions during anaerobic digestion (Li et al., 2021). Consistent with this mechanism, Pan et al. (2019) reported that fruitwood biochar possessed a specific surface area 81 times that of the sawdust biochar adopted in our experiment, and the AD performance of the fruitwood biochar treatment was three times superior to the sawdust biochar group. Such published evidence further supports our conclusion that the greater surface area of corn-stover biochar contributes to its superior cumulative biogas production capacity.

**FIGURE 1:**
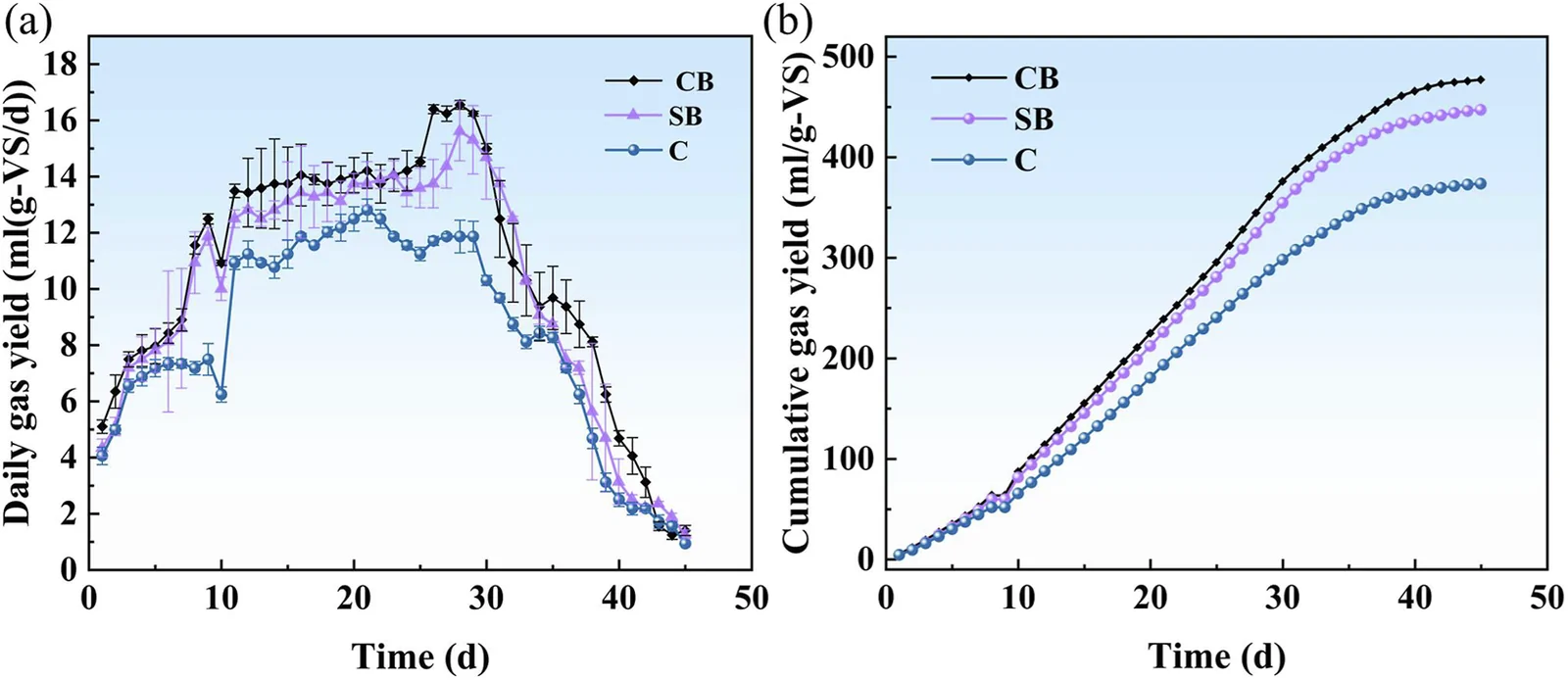
Variations in daily gas yield **(a)** and cumulative gas yield **(b)** during chicken manure anaerobic treatment with adding corn-stover biochar (CB) and sawdust biochar (SB).

Apart from surface structural properties, elemental composition constitutes another key driving factor. As summarized in Supplementary Table S2, corn-stover biochar contains markedly higher concentrations of vital trace and macro nutrients for methanogenic microbes, including Mn, P, K, Mg, Ca, Fe, and Al (Cai et al., 2022; Choong et al., 2016; Pan et al., 2019), which partially accounts for the better biogas performance observed in the CB treatment compared with SB.

#### 3.1.2 Influence of biochar addition on concentration of liquid component

Variations in COD and VFAs reflect the sequential progression of hydrolysis, acidification, acetogenesis, and methanogenesis throughout AD (Chen et al., 2020). During hydrolysis, particulate organic matter is solubilized into COD, which is further bioconverted to VFAs via microorganisms. As illustrated in Figure 2b, the maximum VFA concentrations of the CB and SB groups were 43.4% and 26.2% higher than those of the control, respectively. Conversely, the peak COD contents in the two biochar-amended digesters decreased by 20.3% and 24.8% compared with the control (Figure 2a). This result indicated that biochar addition remarkably accelerated the acidification rate. After day 8 of digestion, the biochar treatments exhibited a more rapid decline in VFA levels alongside persistently lower COD concentrations compared with the control. These observations collectively confirm that both corn-stover biochar and sawdust biochar synergistically stimulate acidogenic, acetogenic, and methanogenic pathways in AD systems.

**FIGURE 2:**
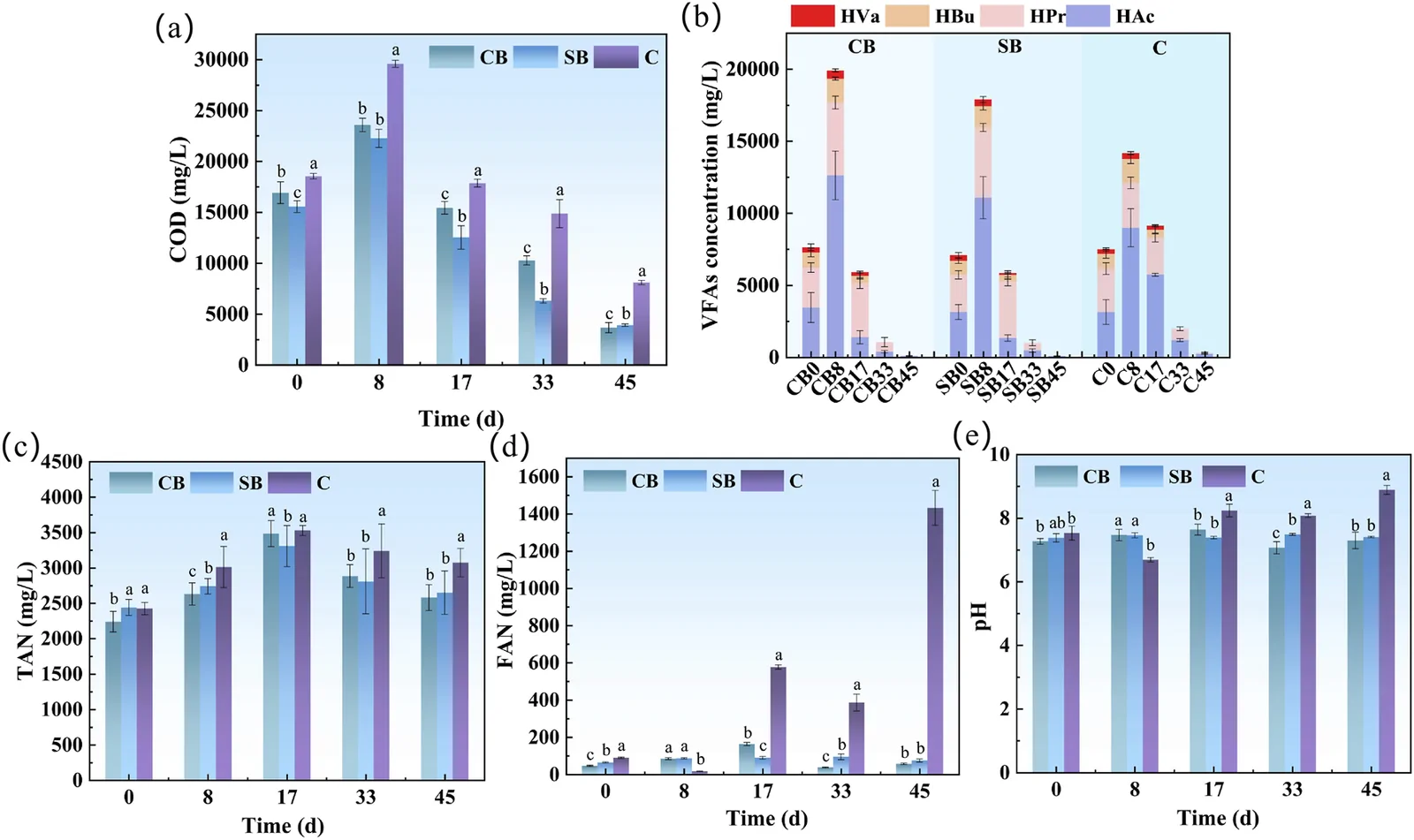
Performance stability including **(a)** COD, **(b)** VFAs, **(c)** TAN, and **(d)** FAN and **(e)** pH of chicken manure AD systems adjusted by different biochars.

From a mechanistic perspective, biochar acts as a conductive carrier that transmits electrons released during VFA oxidation directly to methanogens, substantially improving the efficiency of direct interspecies electron transfer (DIET) (Di et al., 2022). Additionally, morphological analysis (Supplementary Figure S1) reveals that both biochar variants possess abundant porous structures. Such porous frameworks offer extensive surface niches for microbial colonization and proliferation (Zhang et al., 2019). Collectively, biochar addition simultaneously accelerates DIET and microbial biomass accumulation, thereby facilitating all core metabolic stages of AD, namely, hydrolysis, acidogenesis, acetogenesis, and methanogenesis.

#### 3.1.3 Variation of pH relation to daily biogas

During the first 6 days of AD, all three groups exhibited similar trends in daily biogas yield, with the biochar-amended reactors delivering marginally higher biogas output than the control (Figure 1a). From days 7 to 9, the daily biogas production of the biochar treatments rose at a markedly faster rate, and their daily biogas yields exceeded the control by over twofold. In parallel, the pH of the biochar groups remained stable at 7.5, whereas the control pH declined to 6.7 (Figure 2e). The pH of both the biochar treatments and the control fell within the optimal range for AD (6.5–7.5), as documented in prior literature (Li et al., 2022; Zhao K et al., 2024; Pan et al., 2019), which supported the ascending biogas output across all groups.

On day 8, the peak VFA concentrations reached 19,919.1 mg/L (CB), 17,900.0 mg/L (SB) and 14,162.3 mg/L (control), respectively (Figure 2b). The mismatch between VFA generation and VFA degradation created an imbalance between VFA production and consumption, which further triggered massive VFA accumulation in all reactors. However, high endogenous ammonia nitrogen inherently supplied strong alkalinity buffering capacity that neutralizes accumulated acidic metabolites, thus avoiding abrupt, catastrophic pH collapse within the digester (Chen et al., 2008; Zhao J. et al., 2024; Zheng et al., 2021). As listed in Supplementary Table S2, both corn-stover biochar and sawdust biochar were rich in alkali and alkaline earth metals, including Ca, K, Mg, Al, Fe, and Si, which endow the digester with pH buffering capacity (Chiappero et al., 2020). As a result, the control system entered an “inhibited steady state”: it maintained a relatively stable pH level while suffering suppressed biogas productivity and persistent VFA accumulation, which corresponds to a sustainable but low-efficiency, unhealthy operating condition. This property explains why the biochar treatments retained a stable pH of 7.5 even under substantial VFA accumulation. Furthermore, robust biogas generation itself elevates system alkalinity and pH (Jang et al., 2018); accordingly, the superior biogas productivity of biochar groups further stabilized pH against acidification induced by VFA accumulation.

On day 17 of AD, the VFA concentration in all three groups declined, whereas the TAN concentrations increased universally (Figure 2c). The simultaneous reduction of VFAs and elevation of TAN inherently drives pH upward (Li et al., 2022), which contributed to the control’s pH rising to 8.2. In contrast, the pH of biochar-amended digesters remained steadily stabilized at 7.5. After the 17th day, TAN concentrations slightly decreased, VFAs continued to decrease, and biogas production proceeded efficiently. The control’s pH consequently climbed to 8.9, yet the pH of biochar treatments was sustained within the favorable neutral window of 7.0–7.5. This observation confirms that corn-stover and sawdust biochar act as effective pH buffers to maintain neutral digestion conditions.

Notably, the daily biogas production in the control was consistently lower than that of the biochar treatments. Meanwhile, the FAN concentrations of the control remained above 385.7 mg/L, which was remarkably higher than those of the biochar treatments (Figure 2d). The detrimental effects of ammonia inhibition on AD systems can be characterized as a systemic functional disorder. This condition is primarily manifested through impaired microbial metabolic activity, which ultimately leads to a decline in performance and instability of the entire reactor (Cai et al., 2022; Xiao et al., 2019). The tolerance ranges of different AD systems to ammonia nitrogen vary considerably; the inhibition thresholds for TAN and FAN can fluctuate within broad ranges of 1,033–9,000 mg/L and 34.4–200 mg/L, respectively. At the end of digestion, the maximum FAN concentration in the control peaked at 1,432.78 mg/L, whereas FAN levels in biochar treatments stayed below 100.0 mg/L. Consistent tendencies were also observed for soluble COD and VFA profiles, demonstrating that severe FAN toxicity aggravated the inhibited steady state and led to incomplete AD in the control group.

### 3.2 Variation in ARGs and *intI1*

#### 3.2.1 ARGs

Figure 3 illustrated the variations in the absolute abundances of typical macrolide, sulfonamide, and tetracycline ARGs in control, corn-stover biochar (CB), and sawdust biochar (SB) groups throughout anaerobic digestion. Overall, different types of ARGs exhibited distinct response patterns to biochar amendment, and the regulatory effects were highly dependent on biochar types.

**FIGURE 3:**
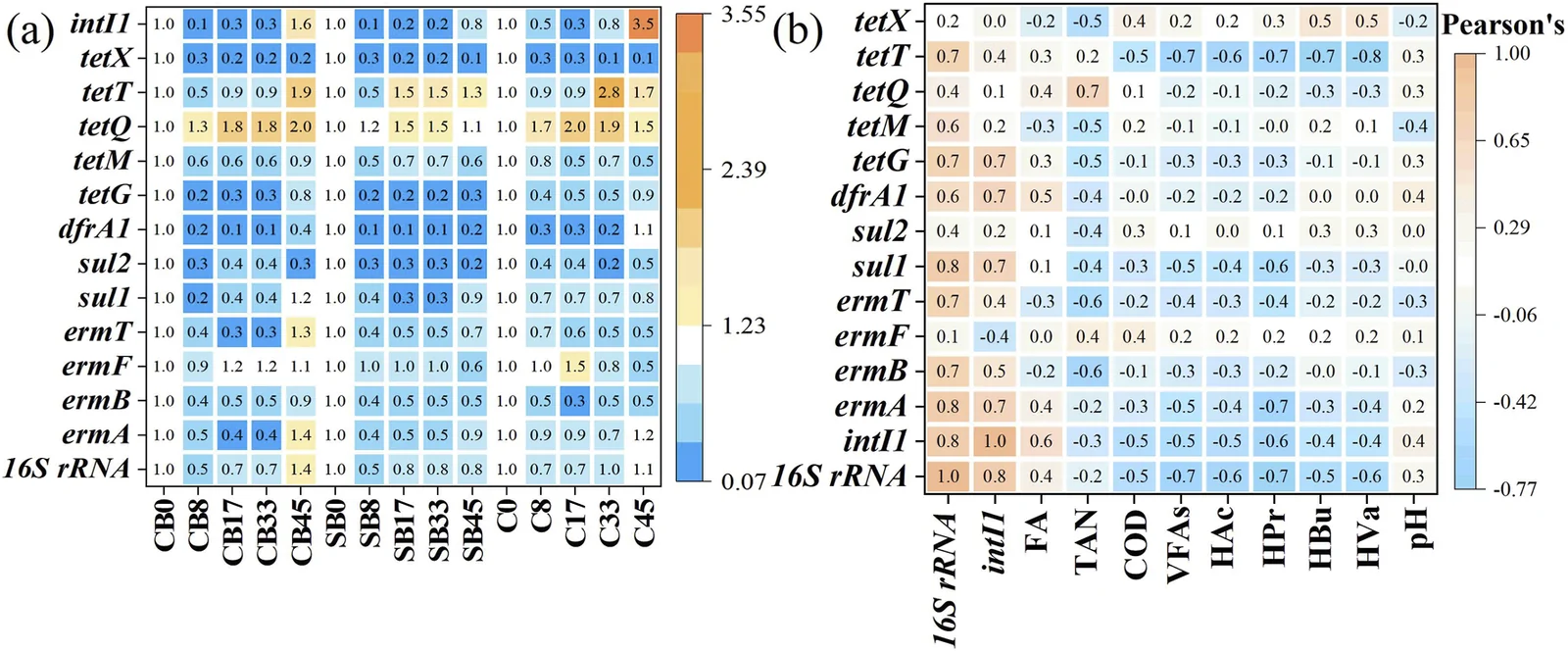
Multiple relative abundances of 16S rRNA, ARGs, and *intI1* compared with day 0 in control and treatment **(a)**, the Pearson’s correlation among ARGs, *intI1*, and environmental factors **(b)**.

For macrolide ARGs, divergent accumulation and removal behaviors were observed among treatments. At the end of AD, the CB group presented elevated abundances of *ermA*, *ermF*, and *ermT*, with 1.4-, 1.1-, and 1.3-fold increases relative to the initial day, respectively, resulting in an overall 8.9% increase in total macrolide ARG abundance. In comparison, the abundances of macrolide ARGs were substantially reduced by 50.4% and 44.6% in the SB and control groups, respectively. This phenomenon demonstrated that sawdust biochar effectively mitigated macrolide ARG proliferation, whereas corn-stover biochar failed to inhibit, and even promoted, the accumulation of macrolide ARGs during AD.

Sulfonamide ARGs showed the most favorable removal performance in the SB treatment. The key sulfonamide subtypes (*sul1* and *dfrA1*) were significantly depleted in the SB group, with their final abundances decreasing to 0.9- and 0.2-fold of the initial, respectively. Correspondingly, the total sulfonamide ARG abundance in the SB group decreased by 61.2%, which was markedly higher than the removal efficiencies of 38.9% and 36.5% in the CB and control groups. Such results verified that sawdust biochar possessed a prominent optimizing effect on sulfonamide ARG elimination, which could be attributed to its strong adsorption capacity and microbial community regulation function.

Tetracycline ARGs displayed subtype-specific variation characteristics after AD. The subtypes *tetG* and *tetM* were universally reduced in all three groups, whereas *tetQ* and *tetX* showed varying degrees of accumulation, indicating that different tetracycline ARG subtypes had different environmental adaptability and proliferation risks under anaerobic conditions. From the perspective of total abundance, the SB group achieved the optimal removal effect with a reduction of 50.8%, whereas the CB group only obtained a low removal rate of 13.9%. Such evident differences further validated that sawdust biochar facilitated the elimination of tetracycline ARGs during AD, whereas corn-stover biochar considerably impaired the ARG removal capacity of the anaerobic system.

In summary, sawdust biochar and corn-stover biochar exerted divergent effects on ARG profiles during anaerobic digestion. Sawdust biochar remarkably reduced the abundances of macrolide and sulfonamide ARGs, whereas corn-stover biochar tended to enrich specific ARG subtypes. Consistent with previous research (Li et al., 2022), biochar with a larger specific surface area exhibited no superiority in eliminating sulfonamide and tetracycline ARGs. Consequently, sawdust biochar with a moderate, smaller specific surface area is more favorable for ARG mitigation during the anaerobic digestion of chicken manure.

#### 3.2.2 Relationship between ARGs, *16S rRNA*, *intI1*, and environmental factors

The relative abundance of *16S rRNA* and *intI1* increased several times in CB and control, whereas it decreased in SB at the end of AD (Figure 3a). The Pearson’s correlation between *16S rRNA* and *intI1* was 0.8 (Figure 3b), implying that sawdust biochar may have suppressed the proliferation of microorganisms carrying intI1, which contributed to the decline of its absolute abundance. Additionally, the *16S rRNA* and *intI1* were positively correlated with multiple ARGs, such as *ermA*, *ermB*, *ermF*, *sul1*, *dfrA1* and *tetG*, and *tetT* and *tetM*, which were only positively correlated with *16S rRNA.* Meanwhile, the absolute abundance of macrolide and sulfonamide ARGs removal was boosted in AD with sawdust biochar, implying that additional sawdust biochar improved ARG removal by reducing the *16S rRNA* and *intI1* abundance.

Moreover, Pearson’s correlation revealed that the *intI1* abundance was positively correlated with FAN, and *tetQ* abundance was positive correlated with TAN. This result indicates that FAN and TAN were the main environmental factors affecting ARG removal. However, the FAN and TAN content in CB and SB were consistently inferior to the control, indicating that FAN and TAN have no direct effect on ARG removal. It is also necessary to continue to analyze the primary reason why sawdust biochar added AD showed excellent ARG removal.

### 3.3 Microbial communities

#### 3.3.1 Microbial communities associated with AD stability

The succession of bacterial phyla communities in three groups is shown in Figure 4a. The dominant bacterial phyla were *Firmicutes*, *Bacteroidetes*, *Spirochaetes*, and *Proteobacteria* in the three groups, which widely appeared in chicken manure AD (Lee et al., 2015; Zhang et al., 2019). Interestingly, the relative abundance of *Spirochaetes* rose first and then fell in all three groups, and the relative abundance of *Spirochaetes* was higher in the biochar treatment than in the control at the end of AD. *Spirochaetes* could participate in syntrophic acetate oxidation in AD with higher TAN, promoting the methanogenesis process (Lee et al., 2015). This may be the reason for the highest cumulative biogas production in AD with supplementation of corn-stover biochar and sawdust biochar.

**FIGURE 4:**
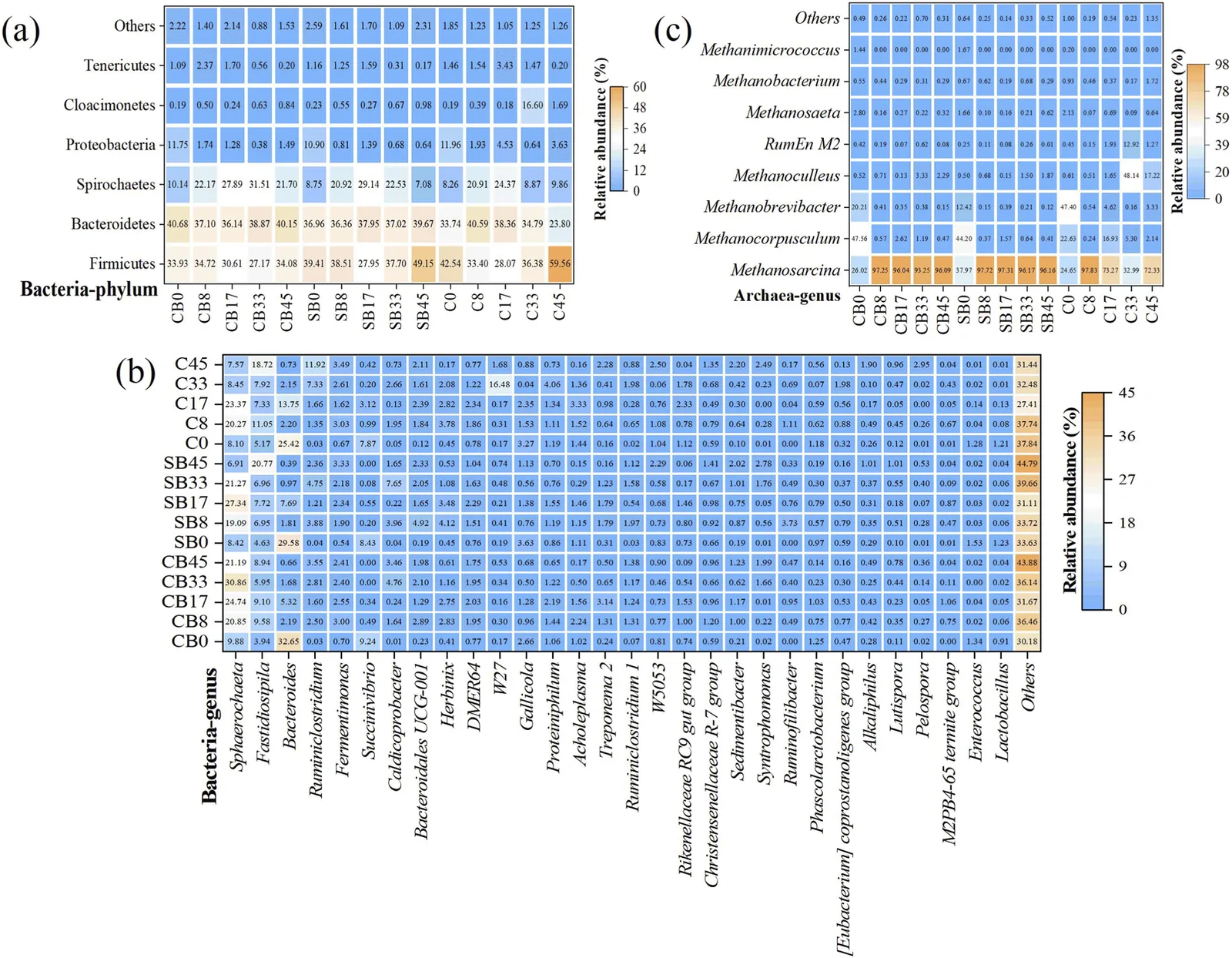
Bacteria communities change at the phylum level **(a)** and genus level **(b)** and archaeal community changes at the genus level **(c)** with a relative abundance over 1.0% during chicken manure AD in the CB, SB, and C.

The profile of bacteria with relative abundance higher than 1.0% at the genus level is shown in Figure 4b. On day 45 of AD, the relative abundance of *Fastidiosipila* was 8.94%, 20.77%, and 18.72% in CB45, SB45, and C45, respectively, which were all increased compared with day 0 (5.17%). *Fastidiosipila* mainly contributes to hydrolytic acidification to produce HAc and HBu as substrates for methanogens during AD (Kim et al., 2018; Pan et al., 2019). Similarly, *Sphaerochaeta* is likewise involved in the hydrolytic acidification of AD (Lü et al., 2016; Zhang et al., 2018), and its relative abundance increased to 21.19% in CB but decreased to 6.91% and 7.57% in SB and control after AD, respectively. In addition, the relative abundance of *Ruminiclostridium* and *Caldicoprobacter* increased after AD operation. One is that the relative abundance of *Ruminiclostridium* was 11.92% in control, which was higher than in CB (3.55%) and SB (2.36%). Another is that the relative abundance of *Caldicoprobacter* was 3.46% in CB, and higher than in SB (1.65%) and Control (0.73%). Some studies have reported that *Ruminiclostridium* and *Caldicoprobacter* could degrade complex cellulose (Fernandez-Gonzalez et al., 2019; Jensen et al., 2021; Ziganshina et al., 2017). Notably, *Ruminiclostridium* was positively correlated with FAN (Figure 5) and grew well in AD with high FAN concentrations (Fernandez-Gonzalez et al., 2019). Furthermore, FAN was significantly higher in AD without biochar than in AD with biochar in this study. It can be speculated that biochar addition reduced the relative abundance of *Ruminiclostridium* by reducing the FAN concentration. In a word, biochar addition increased the *Caldicoprobacter* abundance but decreased *Ruminiclostridium* abundance, and the corn-stover biochar favored the growth of *Sphaerochaeta* while sawdust biochar favored the growth of *Fastidiosipila.*

**FIGURE 5:**
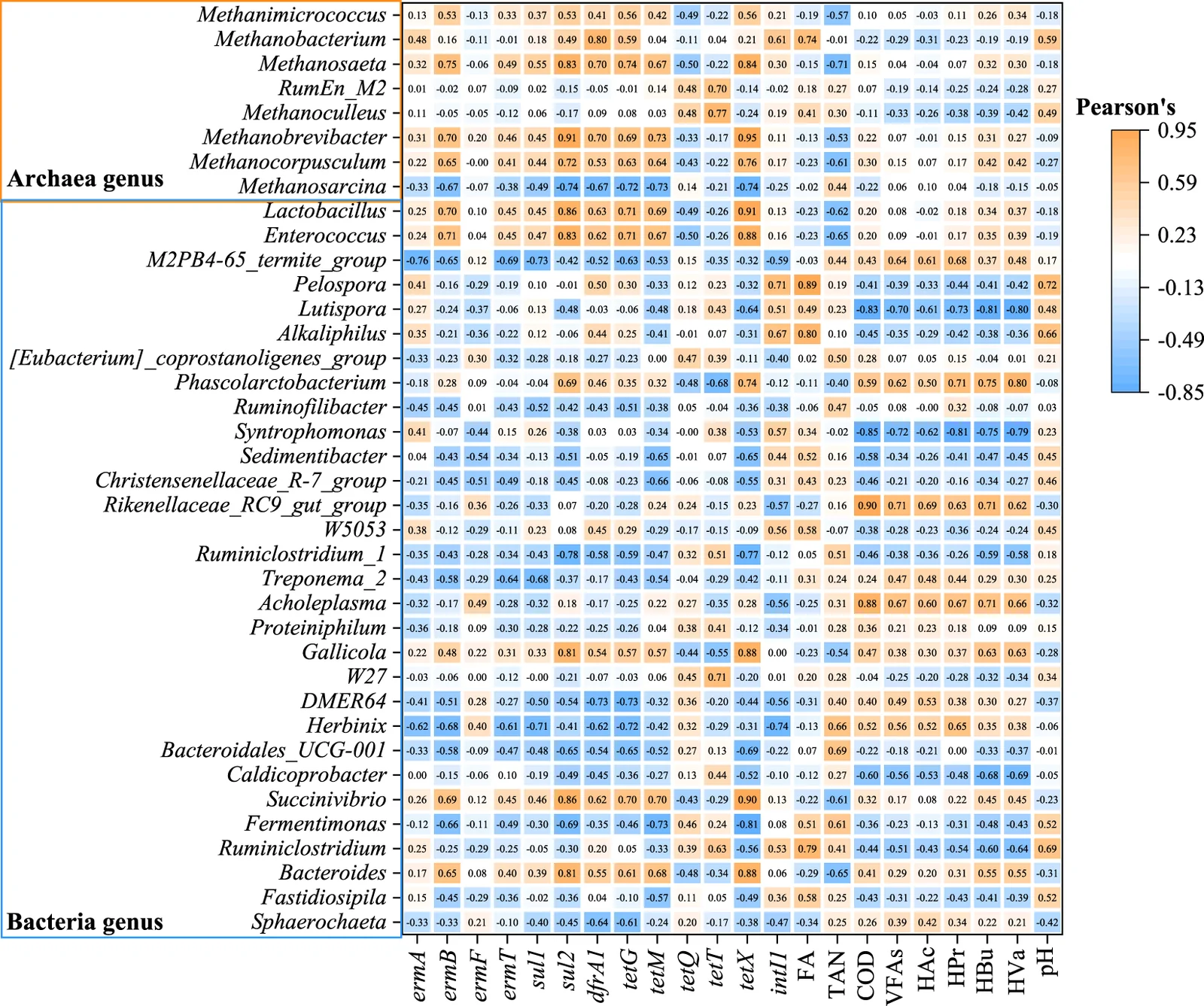
Pearson’s correlation between ARGs, environmental factors, and bacterial/archaea genera with a relative abundance over 1.0%.

The variation of archaeal communities at the genus level is shown in Figure 4c. Compared with day 0 of AD, the relative abundances of *Methanosarcina* increased to 96.09% and 96.16% in CB and SB, respectively, which were higher than in the control (72.33%). Adding biochar enabled the AD system to enrich archaea (i.e., *Methanosarcina*) capable of utilizing multiple nutrients, improving biogas production (Lü et al., 2016). In addition, *Methanosarcina* could co-occur with *Sphaerochaeta* to achieve direct electron transfer in AD, accelerating biogas production (Li et al., 2020). Therefore, corn-stover biochar was more efficient in improving gas production than sawdust biochar in this study.

#### 3.3.2 Genus associated with ARGs

At the genus level, the relative abundance of *Bacteroides* and *Succinivibrio* consistently decreased in the three groups after AD. Pearson’s correlation revealed that *Bacteroides* and *Succinivibrio* were positively correlated with *ermB*, *ermT*, *sul1*, *sul2*, *dfrA1*, *tetG*, *tetM*, and *tetX* (Figure 5). Similarly, Pearson’s correlation analysis revealed that the relative abundance of *Callicola*, *Enterococcus*, and *Lactobacillus* was positively correlated with *ermB*, *ermT*, *sul1*, *sul2*, *dfrA1*, *tetG*, *tetM*, and *tetX* abundance. Moreover, *Ruminiclostridium*, *W27*, and *Ruminiclostridium 1* were positively correlated with *tetT*. Furthermore, the relative abundance of *Ruminiclostridium*, *W5053*, *Syntrophomonas*, *Pelospora*, *Lutispora*, and *Alkaliphilus* was positively correlated with *intI1*.

Regarding archaeal genera, the relative abundances of *Methanobrevibacter* and *Methanocorpusculum* decreased in the control during AD from 47.40% (C0) to 3.33% (C45) and from 22.62% (C0) to 2.14% (C45), respectively. The relative abundances of both archaeal genera were lower than 0.50% in the biochar addition groups. These two archaeal genera were positively correlated with *ermB*, *ermT*, *sul1*, *sul2*, *dfrA1*, *tetG*, *tetM*, and *tetX* (Figure 5). Meanwhile, the relative abundances of *Methanosaeta* and *Methanimicrococcus* were also positively correlated with *ermB*, *ermT*, *sul1*, *sul2*, *dfrA1*, *tetG*, *tetM*, and *tetX*, and *Methanobacterium* was positively correlated with *ermA*, *sul2*, *dfrA1*, and *tetG*. Moreover, the relative abundance of *Methanobacterium* was positively correlated with *intI1*, and *Methanoculleus* and *RumEn M2* were positively correlated with *tetT*.

### 3.4 Mechanism of biochar-enhanced reduction of ARG abundance

It is generally accepted that shifts in microbial community structure are closely associated with variations in the relative abundance of ARGs (Li et al., 2022; Yang et al., 2021). In this study, the potential hosts can be divided into three categories: One is the multiple-ARG host (*ermB*, *ermT*, *sul1*, *sul2, dfrA1*, *tetG*, *tetM*, and *tetX*) (Figures 6a,b), the second one is the specific host of *tetT* (Figures 6c,d), and the third one is the host of the mobile genetic element *intI1* (Figures 6e,f). The abundance of these hosts in the biochar treatments was lower than that in the control. This result suggests that biochar addition reduced the relative abundance of potential hosts. However, only the absolute abundance of macrolide and sulfonamide decreased in AD with sawdust biochar. The lower SSA of sawdust biochar (2.46 m^2^/g) likely provided limited colonization niches, which could have constrained the proliferation of ARG-carrying microorganisms. In contrast, corn-stover biochar’s larger SSA (44.85 m^2^/g) promoted extensive microbial colonization, inadvertently amplifying the absolute ARG pool despite shifts in community composition. Additionally, both biochars may have enhanced the antioxidant capacity of the AD system, thereby inhibiting the horizontal transfer of ARGs (Yang et al., 2022a), which could partially account for the reduced ARG abundance in the SB group.

**FIGURE 6:**
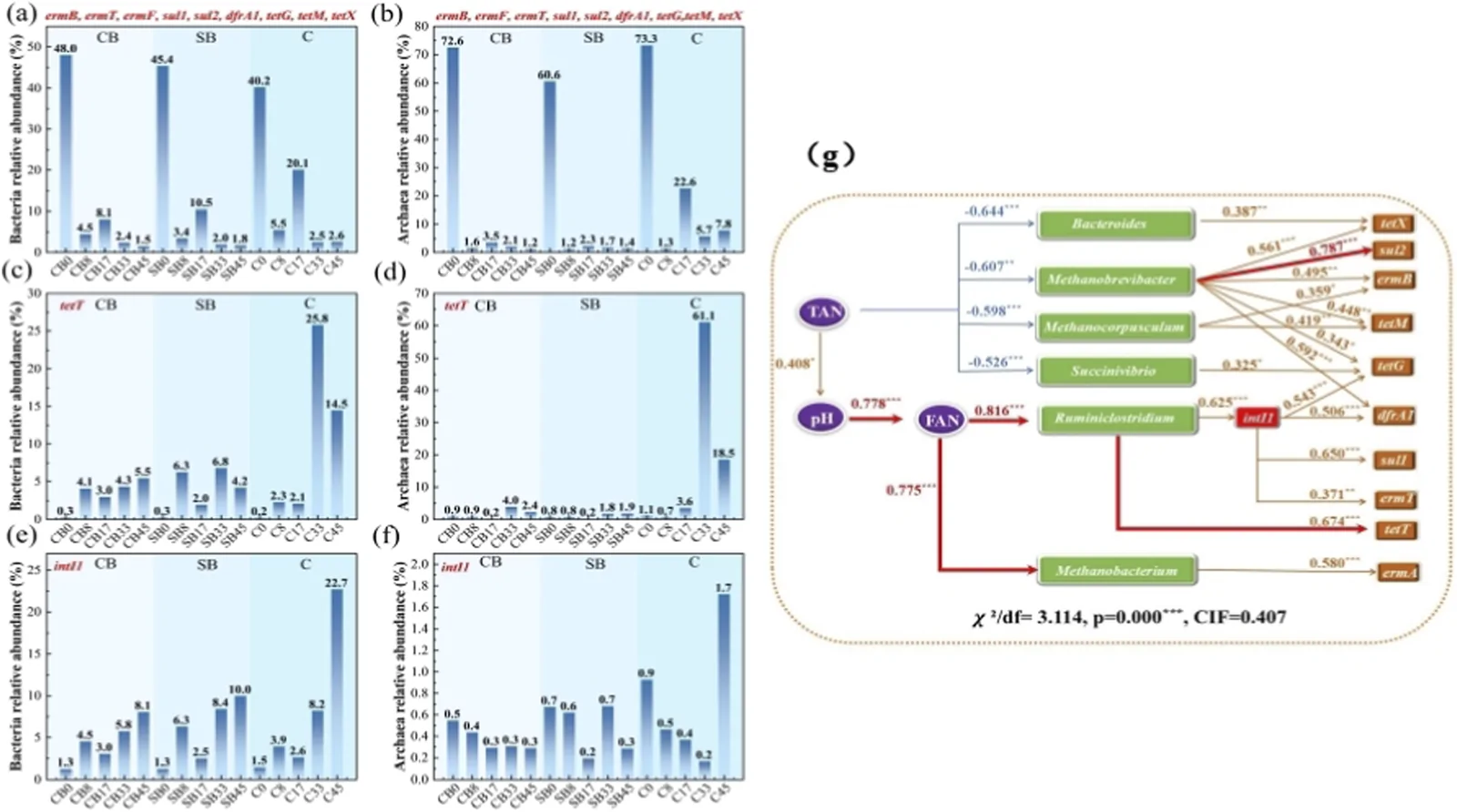
The relative abundance of *ermB*, *ermT, sul1*, *sul2*, *dfrA1*, *tetG*, *tetM*, and *tetX* potential bacterial hosts **(a)** and archaeal hosts **(b)**, *tetT* potential bacterial hosts **(c)** and archaeal hosts **(d)**, and *intI1* potential bacterial hosts **(e)** and archaeal hosts **(f)**, as well as structural equation models (SEMs) **(g)** showing the direct and indirect effects of TAN, pH, FAN, and potential hosts on the ARGs.

In addition, on the basis of the correlation results shown in Figures 3b, 6, environmental factors, ARGs, and potential hosts were selected to establish the SEM. Significant correlations of potential hosts, ARGs, and environmental factors are retained in Figure 6b. TAN, pH, and FAN were the main environmental factors that play a role in the decrease of ARG abundance. TAN imposed an extremely significant negative effect on potential hosts, such as *Bacteroides*, *Succinivibrio*, *Methanobrevibacter*, and *Methanocorpusculum*. In contrast, the FAN concentration has a directed positive effect on the relative abundance of *Ruminiclostridium and Methanobacterium*, which has an indirect effect on the absolute abundance of *intI1* and ARGs. Furthermore, the pH level determines the FAN concentration. However, pH, TAN, and FAN were consistent in the two biochars, indicating that it is not the main reason for the enhanced removal of ARGs in AD with sawdust biochar.

Heredity and transfer occur in microorganisms that are subjected to stress (Li et al., 2022). Additionally, corn-stover biochar and sawdust biochar are conducive to enhance the antioxidant capacity (Supplementary Figure S2) and inhibiting the horizontal transfer of ARGs (Yang et al., 2022b), which may be a reason for sawdust biochar-enhanced ARG removal. In addition, the smaller SSA of sawdust biochar reduced the 16S rRNA absolute abundance (Qin et al., 2020), thus decreasing the *intI1* abundance, which is beneficial for multiple ARG removal. However, the large specific surface area of biochar facilitated multiple microbial growth (Yang et al., 2021), which contributes to excessive reproduction of microorganisms and an increase in ARGs in AD supplemented with corn-stover biochar.

## 4 Conclusion

Corn-stover biochar and sawdust biochar functioned as pH buffers to maintain a neutral pH range of 7.0–7.5. This stabilization mitigated adverse reactions caused by the accumulation of VFAs and FAN, thus facilitating a 20% increase in cumulative biogas production from AD of chicken manure. Notably, sawdust biochar significantly enhanced the removal of macrolide and sulfonamide ARGs, whereas corn-stover biochar tended to enrich specific ARG subtypes. Microbial community succession contributed to the increased biogas production but did not directly govern the reduction of absolute ARG abundance. Instead, the lower specific surface area of sawdust biochar limited total microbial colonization (as reflected by 16S rRNA abundance), thereby reducing the absolute ARG pool more effectively than corn-stover biochar. Collectively, sawdust biochar better balances biogas production improvement and ARG attenuation, exhibiting greater potential for optimizing chicken manure anaerobic digestion than corn-stover biochar. These findings confirm that appropriate biochar selection can simultaneously promote bioenergy recovery and mitigate environmental risks, offering a scalable and cost-effective strategy for sustainable livestock waste management.

This study confirmed that appropriate biochar addition can simultaneously promote biogas yield and mitigate ARG accumulation, resolving the potential trade-off between energy production and environmental risk. From a commercial perspective, sawdust biochar and corn-stover biochar are low-cost, renewable agricultural wastes with wide accessibility. The dual-functional advantages of biochar-stabilizing anaerobic digestion efficiency, improving energy recovery, and reducing ARG pollution endow this modification strategy with excellent practical scalability.

## Data Availability

The data presented in the study are deposited in the NCBI Sequence Read Archive (SRA) repository, accession numbers PRJNA15339936 (BioProject), SAMN63422327-SAMN63422341 (BioSample), and SRR40813751-SRR40813765 (SRA).
