# Supplementary material for: Simultaneous enhancement of biogas production and antibiotic resistance gene removal by sawdust biochar in anaerobic digestion of chicken manure
Source: Front Bioeng Biotechnol. 2026 Oct 21;14:1897972. doi: 10.3389/fbioe.2026.1897972 (PMC13731404; doi:10.3389/fbioe.2026.1897972)
Supplement: Supplementary file 1 [file Supplementaryfile1.docx]

*Supporting Information*

**Simultaneous enhancement of biogas production and antibiotic resistance genes removal by sawdust biochar in anaerobic digestion of chicken manure**

Xiaomei Jiang ^a^, Ting Shen ^a, b,c^*,Yunhui Lei ^a, b, c^ , Xiaobo Guo^a, b, c^

^a^*Biogas Institute of Ministry of Agriculture and Rural Affairs, Chengdu, China,*

^b^*Key Laboratory of Development and Application of Rural Renewable Energy, Ministry of Agriculture and Rural Affairs, Chengdu, China,*

^c^*Research Center for Rural Energy and Ecology, Chinese Academy of Agricultural Sciences, Chengdu, China*

*Corresponding author.

E-mail address: shenting@caas.cn (T. Shen)

| **Gene** | **Forward Primer** | **Reverse Primer** | **Classification** | **Mechanism** |
| --- | --- | --- | --- | --- |
| *16S rRNA* | GGGTTGCGCTCGTTGC | ATGGYTGTCGTCAGCTCGTG | - | - |
| *tetG* | TCAACCATTGCCGATTCGA | TGGCCCGGCAATCATG | Tetracycline | Efflux pump |
| *tetM* | CATCATAGACACGCCAGGACATAT | CGCCATCTTTTGCAGAAATCA | Tetracycline | Cellular protection |
| *tetQ* | CGCCTCAGAAGTAAGTTCATACACTAAG | TCGTTCATGCGGATATTATCAGAAT | Tetracycline | Cellular protection |
| *tetT* | CCATATAGAGGTTCCACCAAATCC | TGACCCTATTGGTAGTGGTTCTATTG | Tetracycline | Cellular protection |
| *tetX* | AAATTTGTTACCGACACGGAAGTT | CATAGCTGAAAAAATCCAGGACAGTT | Tetracycline | other/unknown |
| *ermA* | TTGAGAAGGGATTTGCGAAAAG | ATATCCATCTCCACCATTAATAGTAAACC | Macrolide | Cellular protection |
| *ermB* | TAAAGGGCATTTAACGACGAAACT | TTTATACCTCTGTTTGTTAGGGAATTGAA | Macrolide | Cellular protection |
| *ermF* | CAGCTTTGGTTGAACATTTACGAA | AAATTCCTAAAATCACAACCGACAA | Macrolide | Cellular protection |
| *ermT* | GTTCACTAGCACTATTTTTAATGACAGAAGT | GAAGGGTGTCTTTTTAATACAATTAACGA | Macrolide | Cellular protection |
| *dfrA1* | GGAATGGCCCTGATATTCCA | AGTCTTGCGTCCAACCAACAG | Sulfonamide | Antibiotic deactivate |
| *sul1* | CAGCGCTATGCGCTCAAG | ATCCCGCTGCGCTGAGT | Sulfonamide | Cellular protection |
| *sul2* | TCATCTGCCAAACTCGTCGTTA | GTCAAAGAACGCCGCAATGT | Sulfonamide | Cellular protection |
| *intI1* | CGAACGAGTGGCGGAGGGTG | TACCCGAGAGCTTGGCACCCA | Integron | Integron |

**Table S1** Primer sets used in this study.

| **Table S2 Elemental content of two biochar species.** | | | | | | | | |
| --- | --- | --- | --- | --- | --- | --- | --- | --- |
| Biochar | Si | Al | Fe | Ca | Mg | K | Mn | P |
| Corn-stover biochar | 193.23 | 33.10 | 15.92 | 20.13 | 14.24 | 23.46 | 0.65 | 12.94 |
| Sawdust biochar | – | 0.097 | 0.168 | 2.290 | 0.348 | 1.189 | 0.009 | 0.061 |
| Note: All the unit is g/kg; “–”means below the detection limit. | | | | | | | | |

**Figure S1** Scanning electron micrographs of two biochar: size 100 μm (a) and 10 μm (b) of corn-stover biochar (CB), and size 100 μm (c) and 10 μm (d) of sawdust biochar (SB).

(a)

(b)

(c)

(d)

**Figure S2** The FTIR spectra of corn-stover biochar (CB) and sawdust biochar (SB).
